# Living with a giant, flowering parasite: metabolic differences between *Tetrastigma loheri* Gagnep. (Vitaceae) shoots uninfected and infected with *Rafflesia* (Rafflesiaceae) and potential applications for propagation

**DOI:** 10.1007/s00425-021-03787-x

**Published:** 2021-11-29

**Authors:** Jeanmaire Molina, Dejan Nikolic, Jashvanth Raaj Jeevarathanam, Rinat Abzalimov, Eun-Jung Park, Ronniel Pedales, Elmer-Rico E. Mojica, Danilo Tandang, William McLaughlin, Kyle Wallick, James Adams, Ari Novy, Susan K. Pell, Richard B. van Breemen, John M. Pezzuto

**Affiliations:** 1 Department of Biology, Long Island University, Brooklyn, NY USA; 2 College of Pharmacy, University of Illinois, Chicago, IL USA; 3 Biomolecular Mass Spectrometry Facility, Advanced Science Research Center, City University of New York, New York, NY USA; 4 College of Pharmacy, Long Island University, Brooklyn, NY USA; 5 Institute of Biology, University of the Philippines Diliman, Quezon City, Philippines; 6 Department of Chemistry and Physical Sciences, Dyson College of Arts and Sciences, Pace University, New York, NY USA; 7 Philippine National Herbarium (PNH), Botany Division, National Museum of the Philippines, Manila, Philippines; 8 Academia Sinica, National Taiwan Normal University, Taipei, Taiwan; 9 United States Botanic Garden, Washington, DC USA; 10 San Diego Botanic Garden, Encinitas, CA USA; 11 Department of Anthropology, University of California-San Diego, San Diego, CA USA; 12 Department of Pharmaceutical Sciences, College of Pharmacy, Oregon State University, Corvallis, OR USA; 13 College of Pharmacy and Health Sciences, Western New England University, Springfield, MA USA

**Keywords:** Benzylisoquinoline alkaloid, Ex situ conservation, Holoparasite, LC–MS, Oxylipin

## Abstract

**Main conclusion:**

Metabolites in *Rafflesia*-infected and non-infected *Tetrastigma* were compared which may have applications in *Rafflesia* propagation. Benzylisoquinoline alkaloids, here reported for the first time in Vitaceae, were abundant in non-infected shoots and may be a form of defense. In *Rafflesia*-infected shoots, oxylipins, which mediate immune response, were elevated.

**Abstract:**

Endemic to the forests of Southeast Asia, *Rafflesia* (Rafflesiaceae) is a genus of holoparasitic plants producing the largest flowers in the world, yet completely dependent on its host, the tropical grape vine, *Tetrastigma*. *Rafflesia* species are threatened with extinction, making them an iconic symbol of plant conservation. Thus far, propagation has proved challenging, greatly decreasing efficacy of conservation efforts. This study compared the metabolites in the shoots of *Rafflesia*-infected and non-infected *Tetrastigma loheri* to examine how *Rafflesia* infection affects host metabolomics and elucidate the *Rafflesia* infection process. Results from LC–MS-based untargeted metabolomics analysis showed benzylisoquinoline alkaloids were naturally more abundant in non-infected shoots and are here reported for the first time in the genus *Tetrastigma,* and in the grape family, Vitaceae. These metabolites have been implicated in plant defense mechanisms and may prevent a *Rafflesia* infection. In *Rafflesia*-infected shoots, oxygenated fatty acids, or oxylipins, and a flavonoid, previously shown involved in plant immune response, were significantly elevated. This study provides a preliminary assessment of metabolites that differ between *Rafflesia*-infected and non-infected *Tetrastigma* hosts and may have applications in *Rafflesia* propagation to meet conservation goals.

**Supplementary Information:**

The online version contains supplementary material available at 10.1007/s00425-021-03787-x.

## Introduction

**Fig. 1:**
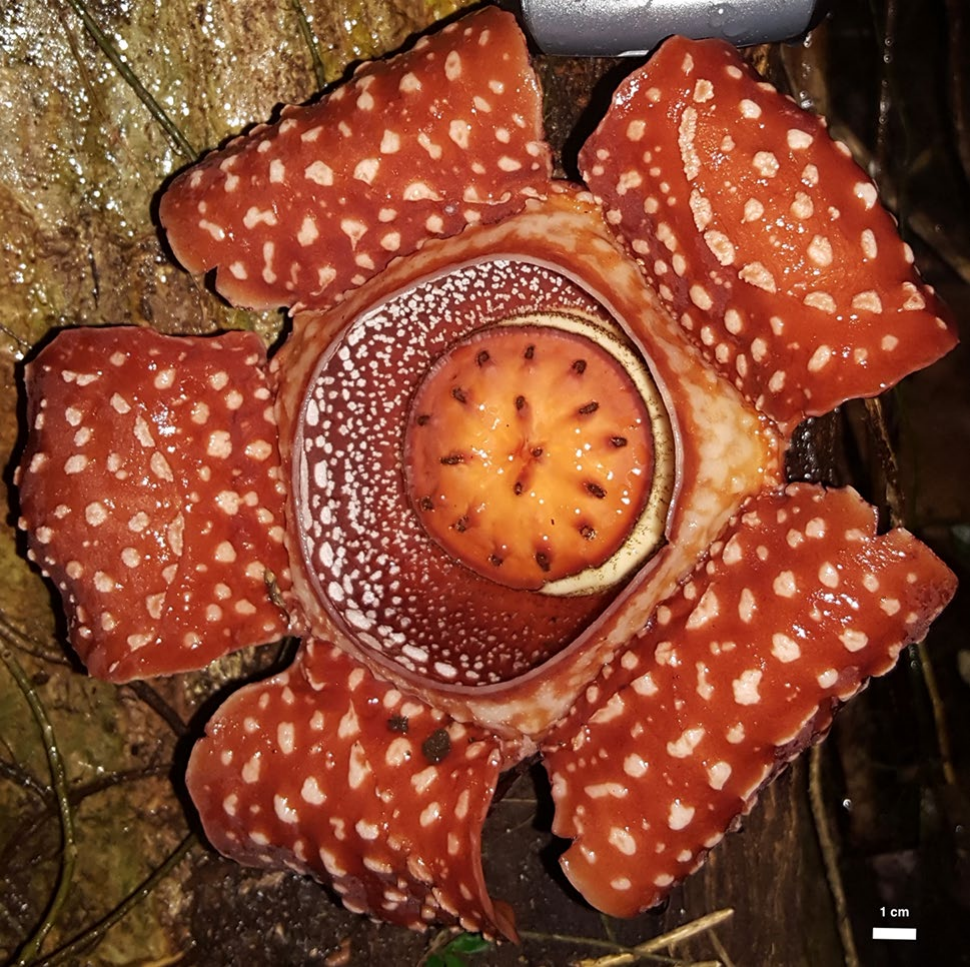
Blooming flower of *R. lagascae* Blanco*,* the *Rafflesia* species infecting *T. loheri* Gagnep. (Mt Guinatungan, Camarines Norte, Philippines)*.* This is one of the smaller *Rafflesia* species, at 20 cm, with the largest *(R. arnoldii* R.Br.*)* growing up to a meter. Photo by J. Molina

*Rafflesia* (Rafflesiaceae, Malpighiales) is a genus of plants producing the world’s largest flowers (Fig. 1), yet it is a holoparasite with no stems, roots or leaves, deriving all nutrients solely from its host vine, the genus *Tetrastigma* (Vitaceae; Nais 2001; Davis et al. 2007). Ironically, the giant-flowered *Rafflesia* produces minuscule threadlike endophytes interspersed within the vascular tissue of its host (Nikolov et al. 2014; Wicaksono et al. 2020). Dubbed the “panda of the plant world” for its charismatic characteristics, *Rafflesia* has ca. 30 species that are unique to the tropics of Southeast Asia with many endangered of extinction (Barcelona et al. 2009; Wicaksono et al. 2016). Unfortunately, efforts to propagate Philippine *Rafflesia* for ex situ conservation have had only incremental success (Molina et al. 2017). Molina and collaborators have been transporting *Rafflesia*-infected *Tetrastigma* cuttings from the Philippines for propagation at the United States Botanic Garden (USBG) in Washington, D.C. since 2015. The cuttings have rooted and survived for a maximum of 11 months, but no shoots have been produced.

Since Indonesia’s Bogor Botanic Garden achieved blooms grafting *Rafflesia*-infected *Tetrastigma* to a *Tetrastigma* rootstock (Mursidawati et al. 2015; Wicaksono et al. 2016), this technique was replicated at the USBG. Unfortunately, the scion did not survive past 33 days. However, transported uninfected *Tetrastigma cf. magnum* Merr. and *Tetrastigma harmandii* Planch. seedlings survived at the USBG (Molina et al. 2017) and resulting mature plants have been repeatedly inoculated with *R. speciosa* seeds since Oct. 2017, but the emergence of *Rafflesia* buds has yet to be observed.

In holoparasitic Orobanchaceae, which includes *Striga* and other agricultural pests, a class of chemicals known as strigolactones produced by their host plants have been found to induce the parasitic plant’s seed germination (Runyon et al. 2009; Smith et al. 2014). After germination, the *Striga* radicle grows toward host roots and forms a haustorium that allows the parasite to attach and obtain nutrients from the host. The haustorium is induced by host-derived small molecules (Albrecht et al. 1999; Saucet and Shirasu 2016) called haustorium-inducing factors (HIF) with 2,6-dimethoxy-*p*-benzoquinone (DMBQ) as the most active HIF for *Striga* (Wada et al. 2019). To determine if strigolactones also induce *Rafflesia* seed germination*,* Molina et al. (2017) incubated *R. speciosa* Barcelona & Fernando seeds in GR24, a synthetic version of strigol. Other plant growth regulators were also investigated. However, none were able to stimulate *Rafflesia* seed germination (Molina et al. 2017). It is still unknown what host metabolites could facilitate a *Rafflesia* infection (Wicaksono et al. 2020), which is proving highly detrimental to conservation efforts.

There are limited studies on the chemical ecology of plant parasite–host interactions (Wink and Witte 1993; Loveys et al. 2001; Lozano-Baena et al. 2007; Runyon et al. 2009; Smith et al. 2009; Clarke et al. 2019; Furlan et al. 2019; Mutuku et al. 2020; Piwowarczyk et al. 2020). Since these interactions are metabolically diverse, involving two species of plants that may share biochemical characteristics (Lozano-Baena et al. 2007), chemical analysis is difficult (Allwood et al. 2008). Most studies of plant parasite–host interaction are on the parasitic taxa of Orobanchaceae and *Cuscuta* (Convolvulaceae) (Clarke et al. 2019; Mutuku et al. 2020). As agricultural pests, there has been a drive to understand their chemical ecology, such as germination stimulants (Runyon et al. 2009), to mitigate the economic impact of crop loss from these plant parasites. Other studies of these taxa (Lozano-Baena et al. 2007; Furlan et al. 2019) have examined the chemistry of host resistance to infection and determined that accumulation of phenolic compounds are toxic and suppressive to the parasite. An interplay between salicylates and jasmonates has also been shown to underlie effective plant defenses against insect herbivores, pathogens, and parasitic plants (Smith et al. 2009). Furlan et al. (2019) examined polyphenol content, which has also been implicated in plant defense, between *Tapirira guianensis* Aubl. trees (Anacardiaceae) parasitized and not parasitized by the mistletoe *Phoradendron perrottetii* (DC.) Eichler (Santalaceae), noting that parasite-infected tissues have less tannin/polyphenol content than healthy tissues.

In this study, we aimed to compare the metabolites in *Rafflesia*-infected and non-infected *Tetrastigma* shoots to understand how *Rafflesia* infection affects host metabolomics. To our knowledge, this is the first study of its kind. A previous study of metabolites of *Tetrastigma hemsleyanum* Diels & Gilg, a medicinal Chinese plant, but not a host of *Rafflesia*, identified constituent flavonoids, anthraquinones, esters, fatty acids, phenols, and catechins (Ding et al. 2019). Another *T. hemsleyanum* study elucidated the regulatory network of anthocyanin biosynthesis including metabolites involved in flavonoid biosynthesis and tryptophan metabolism, as well as alkaloids derived from the shikimate pathway (Yan et al. 2020). Because liquid chromatography–mass spectrometry (LC–MS)-based untargeted metabolomics has proven to be a quick, selective and highly sensitive method of analysis for a wide range of non-volatile metabolites (Commisso et al. 2013; Sargent 2013), we performed LC–MS to compare non-parasitized *Tetrastigma* shoots with infected shoots, to elucidate differences in host chemistry that could identify compounds useful in facilitating *Rafflesia* infection, and consequently, provide a new tool for conservation efforts.

## Materials and methods

Cuttings of *Rafflesia lagascae*-infected *Tetrastigma loheri* Gagnep. and non-infected shoots were collected from San Lorenzo Ruiz Municipality, Mt. Guinatungan, Camarines Norte, Philippines, with permission from Mayor Nelson de Leon in May 2017 and in Aug 2018 (with Gratuitous permit no. 257 and 275 from the Philippine Biodiversity Management Bureau). The non-infected cuttings were taken from sufficiently mature woody host vines that did not have any visible sign of *Rafflesia* infection (i.e. *Rafflesia* floral buds/scars absent), but mature enough that they could presumably support an infection, since *Rafflesia* has never been observed to infect juvenile vines. Our guide Ani Malate, who lived within the vicinity, has been tasked by the local government to regularly monitor *Rafflesia* populations for in situ conservation purposes, and is thoroughly experienced in determining which *Tetrastigma* plants were *Rafflesia-*infected. However, we were limited in sampling to ensure continued natural propagation of the *Rafflesia* and *Tetrastigma* populations and because of physical challenges in accessing various populations interspersed on mountainous terrain.

Samples were kept viable in moist sphagnum moss for about a week after collection during inspections and courier transport to USBG in Washington, DC (with USDA import permit P526P-18-02,136). Upon arrival at USBG, samples were placed in − 80 °C freezer until methanol extraction at LIU-Brooklyn (shipped from USBG overnight in dry ice). Samples from the cuttings were DNA-barcoded to determine the species of *Tetrastigma* following methods described previously (Molina et al. 2018).

Sections within ca. 5 cm of a *Rafflesia* bud, as well as comparable sections from non-infected cuttings (3 individuals for each type of cutting), were subjected to independent liquid chromatography mass spectrometry (LC–MS) experiments for confirmatory analyses, conducted at two different institutions: University of Illinois-Chicago College of Pharmacy (UIC) and the Advanced Science Research Center (ASRC) of the City University of New York. To control for differences due to sampling, comparable samples (i.e., same-age shoots) from infected and uninfected shoots were obtained. In both LC–MS runs, samples were first extracted in methanol (25 mg ground in 700 µL methanol) in July 2017 and in Nov 2018 following field collection. The extracts were evaporated to dryness under a gentle stream of nitrogen and then transported to UIC (July 2017, one set of infected and uninfected samples) and ASRC (Nov 2018, two sets of infected and uninfected samples). Samples were prepared for injection by reconstituting in 0.3 mL (v/v) of 1:1 (v/v) MeOH/water.

At UIC, samples were then analyzed using LC–MS with YMC AQ reverse phase column 2 × 100 mm, 3 µm) and a Waters SYNAPT quadrupole/time-of-flight mass spectrometer operated in positive ion electrospray mode. A linear gradient was used from 10 to 90% acetonitrile in aqueous formic acid over 30 min at a flow rate of 0.2 mL/min (column temperature 30 ºC) with an injection volume of 5 µL. The mass spectrometric data were collected over the range *m/z* 120–900.

At ASRC, samples were analyzed using a Bruker Daltonics maXis-II UHR-ESI-QqTOF mass spectrometer coupled to a Thermo Scientific Ultimate-3000 UHPLC system. Up to 20 µL were injected onto an Agilent Acclaim 120 C_18_-column (2.1 mm × 100 mm, 5 µm) at 30 °C with a flow rate of 200 µL/min. The gradient used was 0–1 min 7% solvent B (acetonitrile, 0.15% formic acid) and 93% solvent A (water, 0.15% formic acid) followed by a gradient 7–35% B from 1 to 15 min, 35–95% B from 15 to 28 min, then held at 95% B from 28 to 31 min. All experimental data were acquired over the range *m/z* 50–1500 using positive ion electrospray. The raw data were analyzed using the online version of XCMS metabolomics software (version 1.10.9; Tautenhahn et al. 2012). XCMS has been developed to facilitate an efficient workflow for untargeted metabolomics, which in contrast to targeted metabolomics, measures as many metabolites in the sample as possible. XCMS integrates metabolite profiling and identification in one step, including peak detection, retention time correction, chromatogram alignment and quantification (Benton et al. 2015). To analyze the data in XCMS, we applied a pairwise comparison between infected and non-infected samples with default parameters for Bruker Q-TOF (ASRC) with “bio-source = plant”.

After XCMS analysis, the difference reports were filtered. XCMS integrates METLIN’s high-resolution tandem mass spectrometry (MS/MS) database, which includes 1 million molecules including lipids, amino acids, carbohydrates, toxins, small peptides, and natural products, among other classes (Guijas et al. 2018). The features from XCMS with *P* value < 0.05, intensities above 20,000, and fold difference of at least 2.5, were analyzed further in Bruker Compass Data Analysis v4.3 and Metfrag Web (Ruttkies et al. 2016; https://msbi.ipb-halle.de/MetFragBeta/) to identify metabolites of interest. These parameters may be arbitrary but careful inspection of aligned peaks showed that these settings clearly distinguished the two groups providing a preliminary assessment of compounds that strongly differed between infected and non-infected hosts. We manually inspected each feature that passed our settings. Those that did not show a pronounced peak difference between the aligned extracted ion chromatograms (EIC) of features in infected and uninfected samples and/or those that were not annotated by METLIN were not included in further analysis.

The neutral molecular formula of the precursor ions (desired features) and their MS/MS fragmentation spectra were then obtained in Bruker Compass Data Analysis and given as input in the MS/MS peak list in Metfrag. All other settings were kept at default values. Candidate metabolites were then retrieved with the highest scoring candidates subjected to additional analysis in CFM-ID (Allen et al. 2014; http://cfmid.wishartlab.com/) to confirm Metfrag candidates. Metfrag and CFM-ID are silico fragmentation tools that utilize known compounds from structure databases to calculate fragments that are matched to experimentally obtained spectra (Blaženović et al. 2018). In addition to these automated approaches, we have also performed a manual dereplication approach on the data obtained from UIC to compare and verify the metabolites of interest from ASRC, as described in previous publications (Gödecke et al. 2009; Nikolić et al. 2012, 2015, 2017), some of which contain extensive analysis of fragmentation spectra of benzylisoquinoline alkaloids (BIA). Tandem mass spectra of BIAs and other metabolites identified in this study were compared to those stored in our in-house library and public databases (MoNA, Horai et al. 2010; GNPS, Wang et al. 2016).

### Data availability

Experimental data from this study are publicly available in NIH Common Fund's National Metabolomics Data Repository (NMDR) website, the Metabolomics Workbench, https://www.metabolomicsworkbench.org, where it has been assigned Project ID PR001169. The data can be accessed directly via Project https://doi.org/10.21228/M8M40V Our data can also be accessed in XCMS including analyses to visualize extracted ion chromatograms (EIC), mass spectra, values of fold change, etc. of relevant features listed in Tables 1 and 2 upon request from corresponding author. In addition, quantitative aspects (fold-change, *P *values, etc.) and corresponding EIC of relevant features presented in Tables S1 and S2 are available in the Supplement.

**Table 1:**
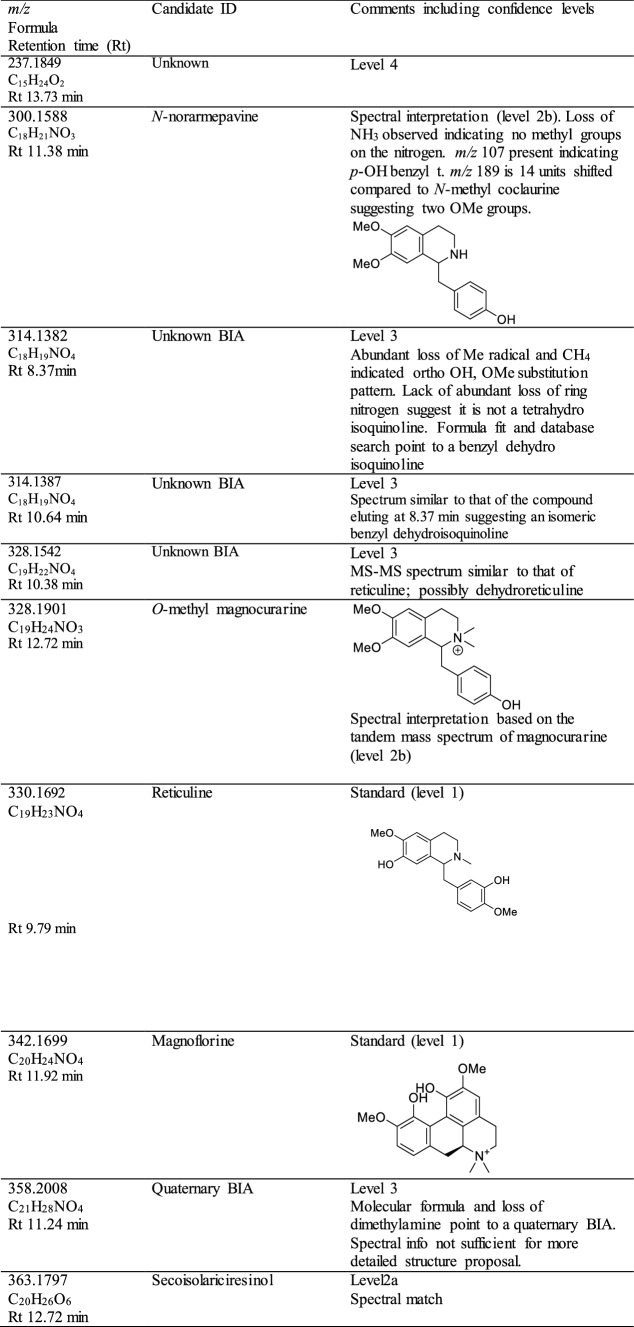
Metabolites significantly elevated in non-infected host. Comments including confidence levels following Schymanski et al. (2014) are provided

**Table 2:**
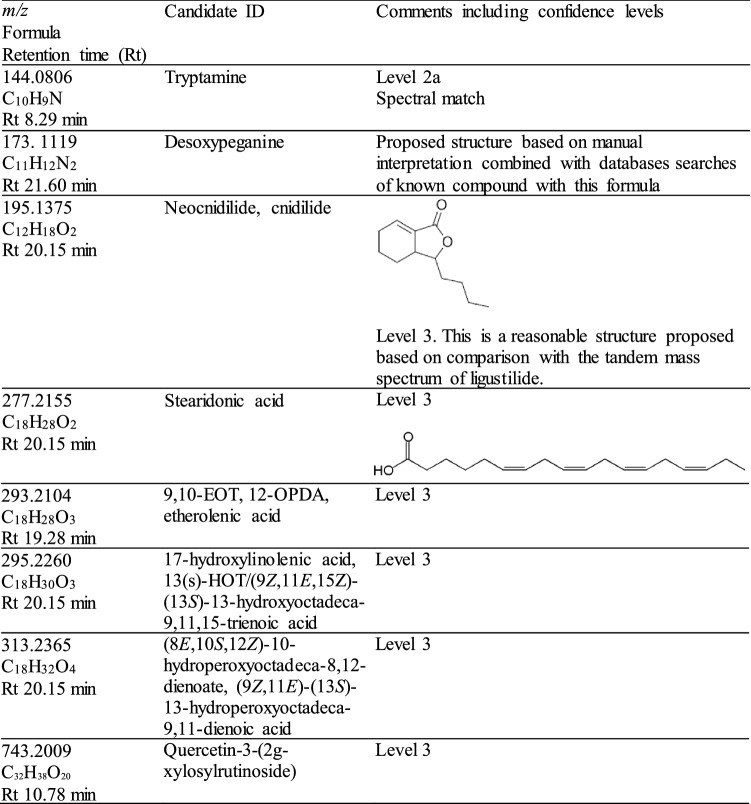
Metabolites significantly elevated in infected host. Comments including confidence levels following Schymanski et al. (2014) are provided

## Results

DNA testing confirmed that 5 of 6 cuttings sampled were *Tetrastigma loheri.* One uninfected cutting was found to be *T. papillosum* (Blume) Planch., and LC–MS raw data for this sample were excluded from further analysis to rule out interspecific differences in metabolites. *T. loheri* is one of the six known *Rafflesia* host species in the Philippines (Pelser et al. 2019).

XCMS analysis yielded 14,457 metabolites/features within the range of retention time 0–35 min. Upon filtering the LC–MS data, there were 422 (out of 14,457) features with *P* values < 0.05, intensities above 20,000, and at least 2.5-fold difference between infected and non-infected *T. loheri*. These settings clearly distinguished the aligned peaks of the two groups providing a preliminary assessment of significantly different metabolites.

Out of the 422 metabolites initially screened, a total of 18 could be further analyzed in Bruker Compass Data analysis, Metfrag and CFM-ID. Metabolites that did not show putative identification in METLIN were excluded. Tables 1 and 2 present metabolites significantly different between infected and non-infected hosts in both LC–MS runs. Since we performed two independent LC–MS analyses of the same samples, we are confident in the detection of these compounds. However, given our limited sampling and the XCMS filters we applied, there may be other compounds we may have missed, and our results are, therefore, preliminary.

**Fig. 2:**
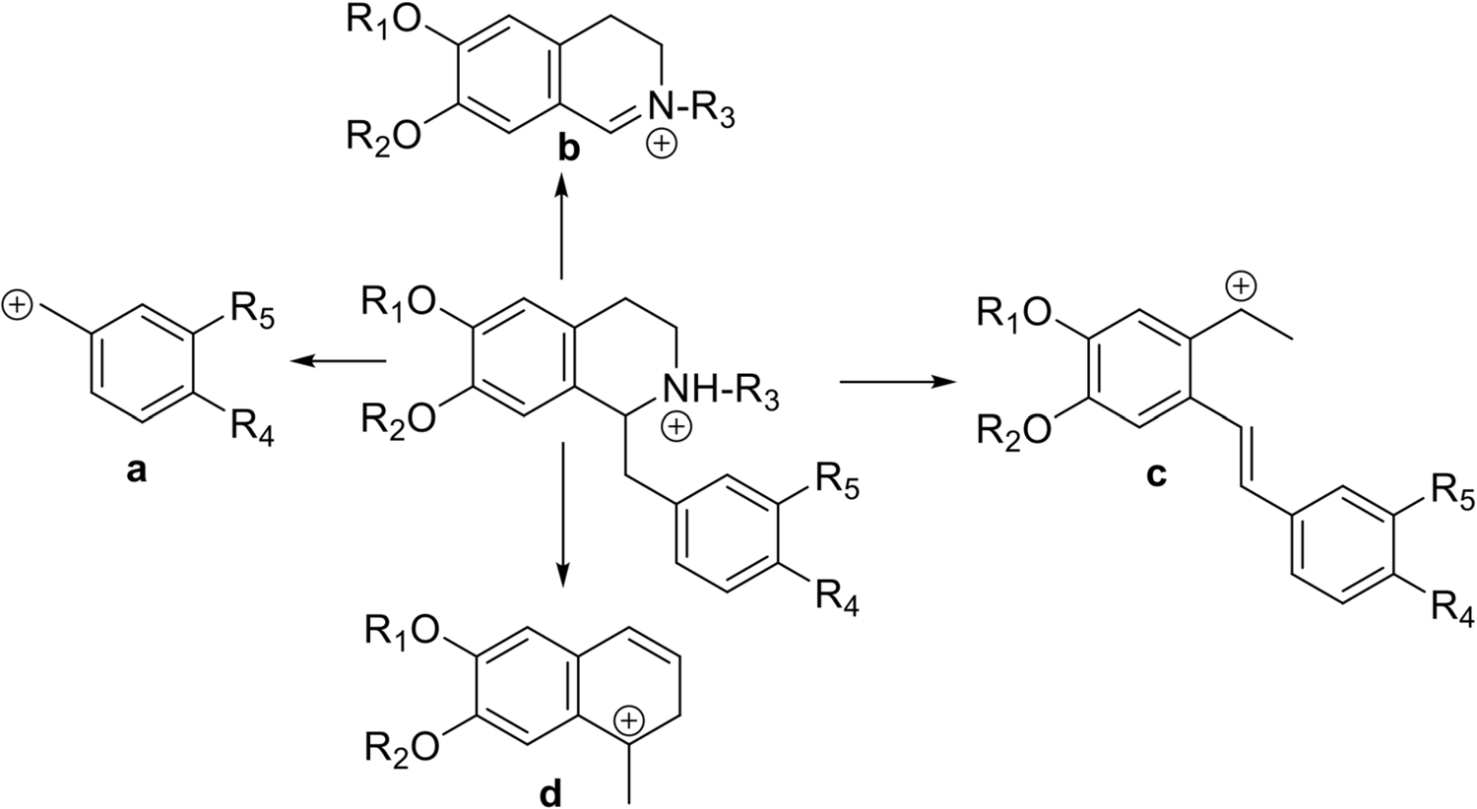
Summary of fragmentation pattern of BIAs. Fragmentation scheme of BIAs used to identify compounds in this study. This summarizes structurally significant fragment ions that can be used to piece together a BIA molecule. Based on the masses of these fragment ions and by comparison with those of the known BIAs one can propose a reasonable structure of the unknown BIAs. For example, ion **c** representing loss of nitrogen determines whether nitrogen is present as a secondary amine (loss of NH_3_), tertiary amine (loss of NH-Me) or a quaternary amine (loss of N(CH_3_)_2_). Quaternary amines have an additional signature ion at *m/z* 58 corresponding to the (CH_3_)_2_ N^+^ = CH_2_ fragment formed by retro Diels–Alder fragmentation. Ion **a** on the other hand determines the substitution pattern on the benzyl side chain. For example, the *m/z* 107 indicates p-OH substituent, while *m/z* 121 indicates p-OMe substituent. The structural arguments used to annotate a specific BIA are shown in Table 1

Nine metabolites belonging to the class of benzylisoquinoline alkaloids were found to be significantly and naturally abundant in the non-infected *T. loheri* cuttings (Table 1). Identification of BIAs was based on the comparison with authentic standards, searches of public and in-house spectral databases and extensive prior knowledge on the fragmentation patterns of these compounds as described in several publications most notably those by Qing et al. (2020) and Menéndez-Perdomo et al. (2021). Figure 2 briefly summarizes structurally significant fragment ions that can be used to piece together a BIA molecule. Based on the masses of these fragment ions and by comparison with those of the known BIAs one can propose a reasonable structure of the unknown BIAs. For example, ion **c** representing loss of nitrogen determines whether nitrogen is present as a secondary amine (loss of NH_3_), tertiary amine (loss of NH-Me) or a quaternary amine (loss of N(CH_3_)_2_). Quaternary amines have an additional signature ion at *m/z* 58 corresponding to the (CH_3_)_2_ N^+^ = CH_2_ fragment formed by retro Diels–Alder fragmentation. Ion **a** on the other hand determines the substitution pattern on the benzyl side chain. For example, the *m/z* 107 indicates p-OH substituent, while *m/z* 121 indicates p-OMe substituent. The structural arguments used to annotate a specific BIA are shown in Table 1.

On the other hand, eight metabolites were found to be significantly elevated in the *Rafflesia*-infected host compared to its non-infected counterpart (Table 2) including tryptamine, desoxypeganine, a pthalide (possibly cnidilide/neocnidilide), various kinds of polyunsaturated fatty acids (PUFA), and a polyphenol.

## Discussion

We sought to determine which metabolites were significantly different between *Rafflesia*-infected and non-infected *T. loheri* to understand how *Rafflesia* infection affects host metabolomics, to characterize these unique metabolites, as well as assess their potential ecological roles to generate insights that can facilitate a *Rafflesia* infection to aid in conservation efforts.

The Orobanchaceae germination stimulant strigol may potentially be present in both infected and uninfected *Tetrastigma* (results not shown) but was not significantly different and, therefore, excluded in the filter. Similar lignin-related compounds that stimulate haustoria in *Phelipanche* and *Striga* (Orobanchaceae, Cui et al. 2018) were also detected in a METLIN search implemented within XCMS, in both *Tetrastigma* samples. However, it is unknown if they are involved in any way in facilitating a *Rafflesia* infection. The absence of germination when *Rafflesia* seeds were exposed to the synthetic strigol GR24 (Molina et al. 2017) suggests that there remain unknown aspects of *Rafflesia’s* germination ecology (Wicaksono et al. 2020).

There were 10 metabolites that were found to be more abundant in non-infected *T. loheri* (Table 1) compared to 8 metabolites in *Rafflesia*-infected shoots (Table 2). Those with known ecophysiological roles in the literature are discussed below. We reiterate that these are compounds that passed our XCMS filters and manual dereplication approach, and there could be other compounds significantly different that have yet to be explored. Nonetheless, our study provides a critical first assessment of compounds that differed between infected and non-infected hosts so we may begin to understand how *Rafflesia* infection affects host metabolomics. It is possible that what we have determined to be “non-infected” hosts may be harboring dormant *Rafflesia* infection (i.e., *Rafflesia* buds have not yet emerged, Bascos et al. 2021), but given our local guide’s expertise and experience in making this determination, and the low chance of unwittingly selecting three “dormant” samples, we are positive of the sampling, though additional sampling and analysis in the future would certainly be advantageous.

### Metabolites abundant in non-infected *Tetrastigma loheri*

Benzylisoquinoline alkaloids (BIA) were significantly detected in non-infected *T. loheri* compared to infected shoots. BIAs are a diverse group of about 2,500 alkaloids that include pharmacologically important drugs such as codeine, morphine, tubocurarine, naturally produced in basal angiosperms (magnoliids including Annonaceae, Lauraceae and Piperales), as well as in the phylogenetically distant angiosperm order Ranunculales and infrequently, in the families Rutaceae, Cornaceae and Nelumbonaceae (Liscombe et al. 2005; Bonamore et al. 2010). BIA biosynthesis may have evolved as a mechanism for plant defense against herbivores and may have originated early on in angiosperm evolution, but its limited occurrence among angiosperms suggests the need for a “highly specialized cellular platform to activate the pathway in divergent taxa” (Liscombe et al. 2005: 2500). BIA is said to be absent in Eudicots (Cole et al. 2019), but our results imply that BIA may be sporadically present in eudicots and can be activated. Isoquinoline alkaloids are synthesized via decarboxylation of tyrosine or DOPA (dihydroxyphenylalanine) to yield dopamine and 4-hydroxyphenylacetaldehyde, which are then metabolized to reticuline, an important precursor of various BIAs. Substitution of the heterocycle isoquinoline at the C1 position by a benzyl group provides 1-benzylisoquinoline (Kanehisa and Goto 2000).

Though the pathway seems to have originated early on in the evolution of angiosperms, BIA production is only active in certain plant groups and is deactivated in others. However, this is the first time these compounds have been reported in *Tetrastigma,* and in the grape family, Vitaceae. As far as we know, no study has found BIA in the model species, *Vitis vinifera* L., the common grape, which has been extensively characterized chemically (Pezzuto 2008; Pinu et al. 2018). However, enzymes involved in the initial steps of the pathway, prior to reticuline, are present in *Vitis* (Kanehisa and Goto 2000).

BIAs identified in non-infected *T. loheri* include reticuline, norarmepavine, magnocurarine, magnoflorine, and a few unknown BIAs (Table 1). In the BIA pathway, (*S*)-reticuline is the most common precursor for most BIAs formed from the methylation of methylcoclaurine. It is unclear how the other alkaloids are sequentially produced. Magnoflorine, magnocurarine have been identified in *Magnolia officinalis* Rehder & E.H.Wilson (Poivre and Duez 2017). Armepavine, the methylated form of norarmepavine, on the other hand, is a major bioactive compound of *Nelumbo nucifera* Gaertn. and has been tested as a potential therapeutic agent for the treatment of a kidney disorder (Ka et al. 2010).

Ironically, much more is known about the pharmacology of the BIAs (Singla et al. 2010), and there is little interest in their ecophysiological roles, which could provide insight as to why uninfected *T. loheri* may be producing these. BIAs generally do not appear essential for plant growth and development, but they play a key role in the plant defense against herbivores and pathogens (Hagel et al. 2013). Thus, in *Tetrastigma loheri*, these chemical adaptations may have evolved to fight off an infection including *Rafflesia*. In *Genista anthoclada* (Fabaceae) and its holoparasite *Cuscuta palaestina* (Convolvulaceae) several quinolizidine alkaloids were detected, with the alkaloids assumed to be exploited by *Cuscuta* for its own antiherbivore protection (Wink and Witte 1993). In addition to BIAs, the lignan, secoisolariciresinol, was also elevated in uninfected shoots. Lignans have potent antimicrobial and insecticidal properties and may have important roles in plant defense (Saleem et al. 2005).

Pennings and Callaway (2002) likened plant parasites to herbivores, as consumers with host preferences, but unlike animal herbivores, plant parasites are immobile and intimate with the host, and are thus more affected by host physiology and host biochemistry. *Cuscuta* seemed to have evolved to resist the toxic effects of the quinolizidine alkaloids and appropriate them for its own defense (Wink and Witte 1993), which does not seem to be the case in *Rafflesia* given that BIAs were found to be lacking in infected shoots. Nonetheless, it is also possible that unidentified microbial endophytes are producing the metabolites identified in *Tetrastigma*, as has been observed in opium poppy and in other plants (Ray et al. 2019).

Alternatively, *Rafflesia* could be suppressing the production of BIA in infected shoots. In terms of its application for ex situ propagation, *T. loheri* shoots with minimal BIA content may be more effective in supporting a *Rafflesia* infection and should be considered when grafting *Rafflesia*-infected shoots and when inoculating *Rafflesia* seeds.

### Metabolites elevated in *Rafflesia*-infected *Tetrastigma loheri*

In *Rafflesia-*infected *Tetrastigma* shoots, there were eight metabolites that were found to be significantly elevated based on our XCMS settings. Noteworthy compounds include tryptamine, a phthalide (possibly cnidilide/neocnidilide) and various kinds of polyunsaturated fatty acids (PUFA) and the polyphenol quercetin-3-(2 g-xylosylrutinoside). The identification confidence for these are at level 3 (except tryptamine at 2a), meaning that the compound class (e.g. oxygenated fatty acids) is known, though the exact ID/structure is unknown (Table 2) and have yet to be elucidated.

In the tryptamine pathway, the amino acid tryptophan is metabolized to tryptamine then ultimately to indole acetic acid, a bioactive auxin (Quittenden et al. 2009). Auxin signaling was found to be important in promoting xylem bridge formation between the Orobanchaceae hemiparasite *Ptheroispermum japonicum* and its host (Wakatake et al. 2020), and we speculate that the elevated trytamine would increase auxin that may forge vascular connections between *Rafflesia* and its host. Desoxypeganine was also found elevated in infected shoots but its ecological role is unknown, though it is pharmacologically characterized as a cholinesterase inhibitor (Algorta et al. 2008). The phthalide cnidilide (or neocnidilide) has been isolated as a flavor constituent in celery oil (MacLeod and Ames 1989). Interestingly, it is structurally related to seed-germination stimulants, strigol and karrikinolide (Renzetti and Fukumoto 2019), possessing a butenolide ring that may be biologically relevant (Fischer et al. 1989). Stearidonic acid is a PUFA naturally found in the seed oils of hemp and flaxseed (Bakowska-Barczak et al. 2020). Stearidonic acid (SDA) is derived from alpha-linolenic acid by a specialized enzyme, delta-6 desaturase, not present in many plants (Ruiz-López et al. 2009). SDA is also important in human nutrition because it is an intermediate in the biosynthesis of eicosapentanoic acid (EPA) and docosahexaenoic (DHA) acids (Whelan et al. 2009). However, such as BIAs, its precise ecophysiological role is unclear, except that it is an intermediate product in the lipid pathways in some plants (Sreedhar et al. 2017). It is interesting to note, however, in the model nematode, *Caenorhabditis elegans,* its inability to produce gamma-linolenic acid and SDA due to loss-of-function mutation in the enzyme delta-6 desaturase led to increased pathogen susceptibility (Nandakumar and Tan 2008). Thus, the release of SDA in *Rafflesia*-infected *T. loheri* may be an immune response.

The PUFA hydroxy linolenic acid and 13(*S*)-HOT (9*Z*,11*E*,15Z)-(13*S*)-13-hydroxyoctadeca-9,11,15-trienoic acid) were also substantially increased in *Rafflesia*-infected shoots. These are types of oxygenated fatty acids, collectively termed ‘oxylipins’, which are involved in the immune response of plants (Genva et al. 2019). The plant hormone and oxylipin, jasmonate, is present ubiquitously in land plants playing a role in defensive responses (Griffiths et al. 2015). Like SDA, jasmonates are formed from linolenic acid in plant chloroplasts. Some oxylipins are distasteful to insect predators, and others can elicit a signal of cell damage throughout the plant to coordinate a comprehensive response (Gessler et al. 2017). Linolenic and linoleic acid production was also found to increase in tomato plants parasitized by the parasitic plant, *Cuscuta pentagona* Engelm.*,* similar to the chemical response tomato plants display when attacked by herbivores or pathogens (Runyon et al. 2010). Fatty acid hydroperoxides (possibly 8*E*,10*S*,12*Z*)-10-hydroperoxyoctadeca-8,12-dienoate and (9*Z*,11*E*)-(13*S*)-13-hydroperoxyoctadeca-9,11-dienoic acid), which serve as important intermediates in the oxylipin pathway (Hamberg et al. 1999), were also detected.

Another type of oxylipin detected in *Rafflesia*-infected shoots was a divinyl ether fatty acid (possibly 12-OPDA or etherolenic acid), which is similarly derived from alpha-linolenic acid from the action of plant lipoxygenases (Fammartino et al. 2007; Vincenti et al. 2019). Though the physiological importance of divinyl ether fatty acids is not fully understood, it was observed that levels of this metabolite increased in infected potato leaves suggesting a possible role in defense response. Based on studies conducted with other plants, it is possible these oxylipins are released by *T. loheri* as a defense mechanism during *Rafflesia* infection. Thus, suppression of oxylipins in *Rafflesia*-infected *Tetrastigma* shoots may be beneficial in facilitating *Rafflesia* propagation.

Phenolics such as flavonoids and tannins have been demonstrated to be involved in plant defense against plant parasites (Lozano-Baena et al. 2007; Furlan et al. 2019). One flavonoid elevated in infected *T. loheri* was quercetin 3-(2 g-xylosyl rutinoside), but its ecological importance is so far not understood, yet multiple studies extol its dietary benefits (Anand David et al. 2016; Salehi et al. 2020). Piwowarczyk et al. (2020) detected abundant polyphenols, such as quercetin derivatives in the host species of the holoparasite *Cistanche armena* (K. Koch) M.V. Agab (Orobanchaceae). In grapes, quercetin glycosides as well as other flavonoids were associated with phytoplasma and mildew infection (Bouderias et al. 2020). Whether these polyphenols are involved in *Tetrastigma* immune reponse against its *Rafflesia* parasite remains to be seen.

## Conclusion

In this study we provided a preliminary assessment of metabolites significantly different between *Rafflesia-*parasitized and non-parasitized *Tetrastigma loheri.* The abundance of benzylisoquinoline alkaloids (BIA) in non-infected host shoots suggest this metabolite may represent a defense strategy against *Rafflesia* infection, or that *Rafflesia* could somehow repress BIA production in infected shoots, where BIAs were shown to be lacking. The presence of BIA, a class of medicinally important compounds, in *Tetrastigma* and in its family Vitaceae, is here reported for the first time and reflects the pharmacological potential of this genus. Secretion of polyunsaturated fatty acids, of oxylipins, and polyphenols in *Rafflesia*-infected shoots, suggest that *Rafflesia* elicits host immune response. Conceivably, suppression of these immune-response compounds could facilitate *Rafflesia* infection and hence propagation. Further studies to test the metabolites identified here are the logical next steps to develop propagation strategies that could prove integral for preservation of this “panda of the plant world.”

## Supplementary Information

Below is the link to the electronic supplementary material.

Supplementary file1 (DOCX 2291 kb)

## Abbreviations

ASRC: Advanced Science Research Center, City University of New York
BIA: Benzylisoquinoline alkaloid
LC–MS: Liquid chromatography–mass spectrometry
PUFA: Polyunsaturated fatty acids
SDA: Stearidonic acid
USBG: United States Botanical Garden
