# Supplementary material for: Living with a giant, flowering parasite: metabolic differences between Tetrastigma loheri Gagnep. (Vitaceae) shoots uninfected and infected with Rafflesia (Rafflesiaceae) and potential applications for propagation
Source: Planta. 2021 Nov 29;255(1):4. doi: 10.1007/s00425-021-03787-x (PMC8627921; doi:10.1007/s00425-021-03787-x)

| fold | *P*-value | updown | mzmed | rtmed | maxint | mean1 (Infected) | sd1 | mean2 (Non-infected) | sd2 |
| --- | --- | --- | --- | --- | --- | --- | --- | --- | --- |
| 24.3403225 | 0.02051302 | UP | 237.184896 | 13.72535 | 57962 | 13664.2689 | 4061.73895 | 332592.711 | 141904.871 |
| 11.324919 | 0.01393538 | UP | 300.158767 | 11.3826083 | 4567084 | 2233911.55 | 1425079 | 25298867.3 | 9114386.28 |
| 24.9412142 | 0.01079867 | UP | 314.138159 | 8.3741 | 2411846 | 645111.496 | 301430.087 | 16089864 | 5454626.67 |
| 5.96842361 | 1.0949E-05 | UP | 314.138655 | 10.6448917 | 193170 | 283664.75 | 213403.254 | 1693031.39 | 168599.583 |
| 16.787233 | 0.00463695 | UP | 328.154215 | 10.3811833 | 155180 | 82117.4618 | 40199.7682 | 1378524.96 | 345835.738 |
| 2.66068883 | 0.00031836 | UP | 328.190055 | 12.7212833 | 299078 | 962244.942 | 239991.043 | 2560234.37 | 328235.272 |
| 3.0124 | 0.04693961 | UP | 330.169159 | 9.7859 | 27554 | 54593.3779 | 20264.6102 | 164457.092 | 69729.1684 |
| 17.2330121 | 7.0805E-05 | UP | 342.169917 | 11.9206 | 65596 | 30630.8912 | 9119.30922 | 527862.519 | 38229.8696 |
| 14.2003185 | 0.0007198 | UP | 358.200844 | 11.2433833 | 90772 | 59181.9811 | 36572.3546 | 840402.979 | 128488.306 |
| 8.77476565 | 0.01290453 | UP | 363.17969 | 12.7235 | 227260 | 125399.953 | 89928.261 | 1100355.2 | 385245.323 |
| 5.17218273 | 0.04563385 | DOWN | 144.080616 | 8.28876667 | 182352 | 1493613.98 | 956616.874 | 288778.27 | 349135.763 |
| 3.84848089 | 0.01053944 | DOWN | 173.111942 | 21.5962583 | 25632 | 134258.231 | 52937.7306 | 34886.0328 | 19227.4793 |
| 8.47900486 | 0.02430693 | DOWN | 195.137495 | 20.14775 | 1130926 | 4325923.2 | 2422901.14 | 510192.325 | 112407.477 |
| 6.9132446 | 0.02717449 | DOWN | 277.215453 | 20.14775 | 784302 | 3064198.73 | 1728690.05 | 443235.977 | 143041.246 |
| 5.23159431 | 0.00887655 | DOWN | 293.210432 | 19.2783167 | 622810 | 3348321.87 | 1284746.97 | 640019.403 | 127100.31 |
| 6.5261233 | 0.0198803 | DOWN | 295.225962 | 20.14775 | 2059344 | 8559572.82 | 4335915.69 | 1311586.13 | 330234.779 |
| 5.77495921 | 0.01994219 | DOWN | 313.236538 | 20.14775 | 619080 | 2898744.91 | 1437936.58 | 501950.715 | 132788.048 |
| 5.795981 | 0.03438028 | DOWN | 743.200943 | 10.77538 | 286362 | 1109312.13 | 651443.9665 | 191393.3463 | 29493.5937 |

**Table**  **S1**(Supplement) Quantitative aspects from XCMS analysis of features presented in Tables 1 and 2. Extracted ion chromatograms (EIC) of these features from are also presented below (**Table S2**).

**Table S2**

EICs: Upregulated in Non-infected Samples (Red) but downregulated in *Rafflesia*-infected samples (black):


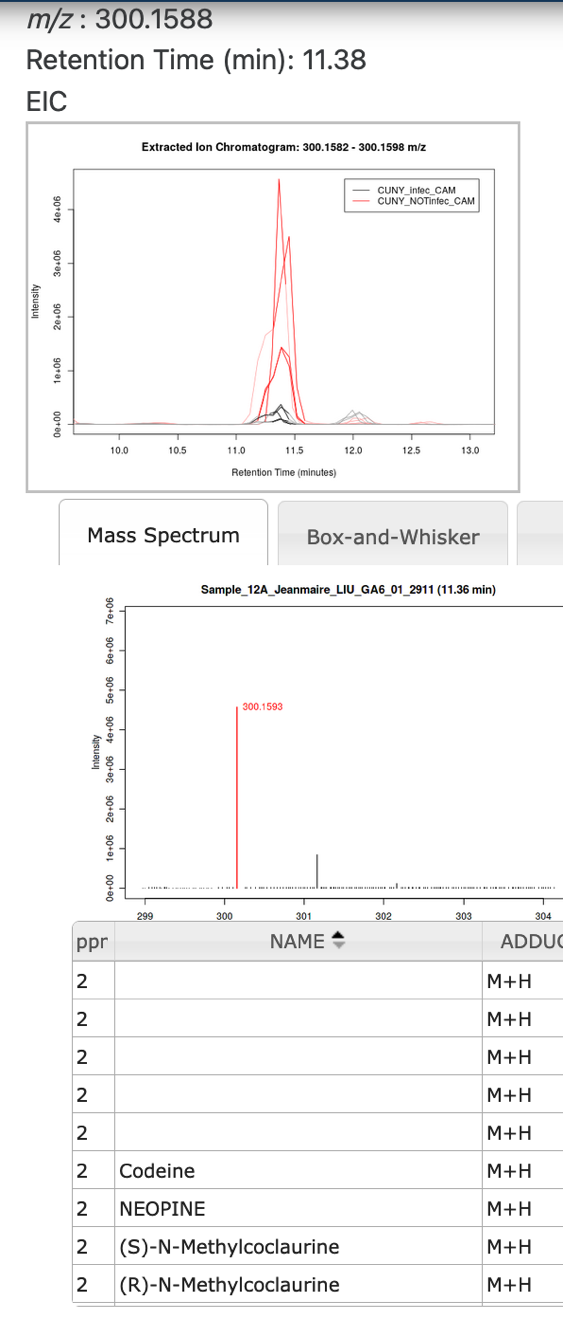

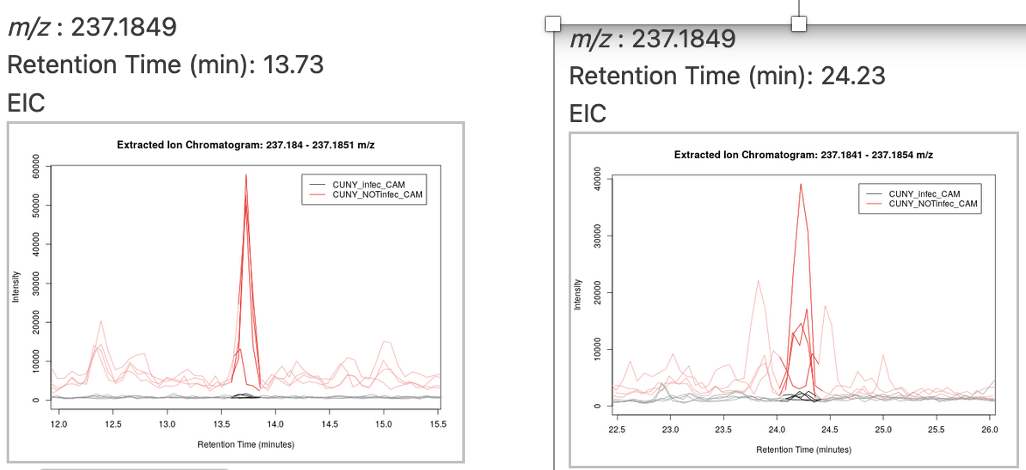

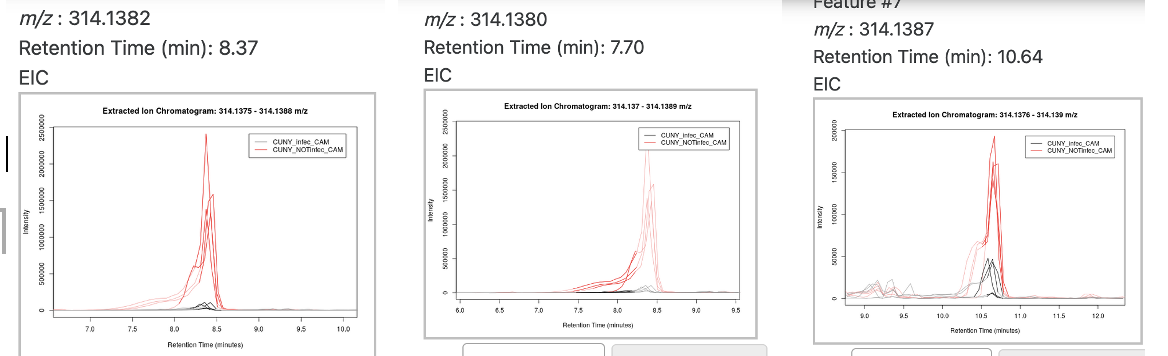


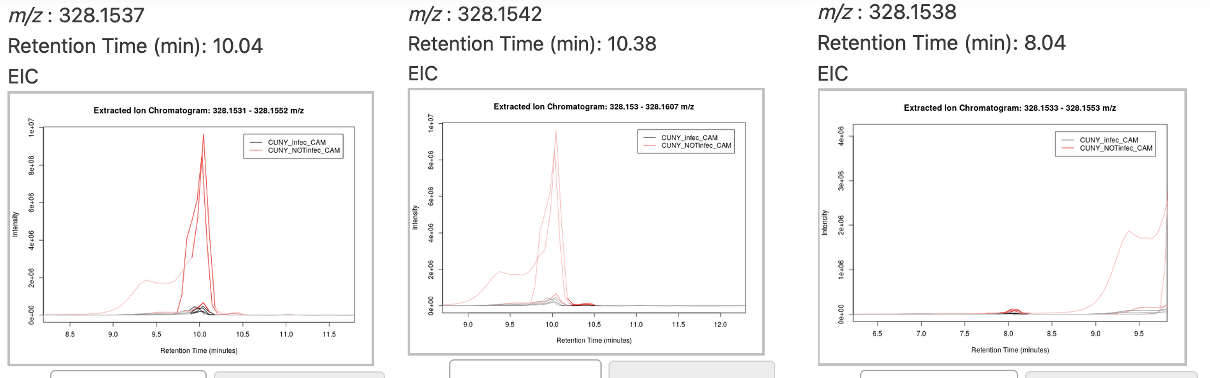


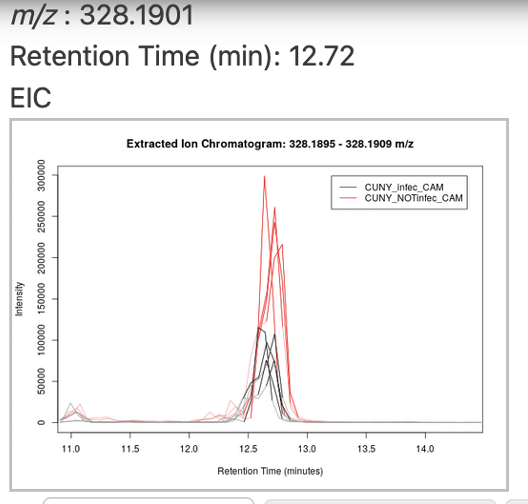

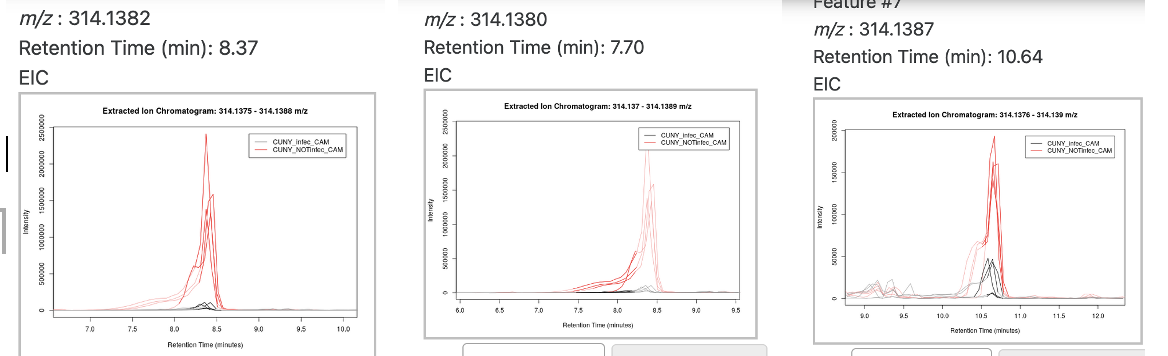


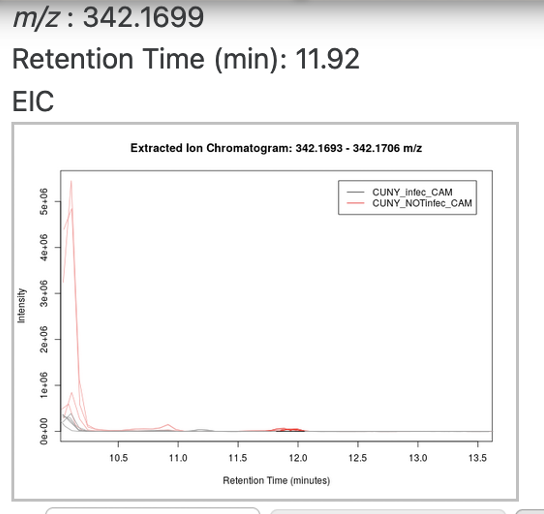

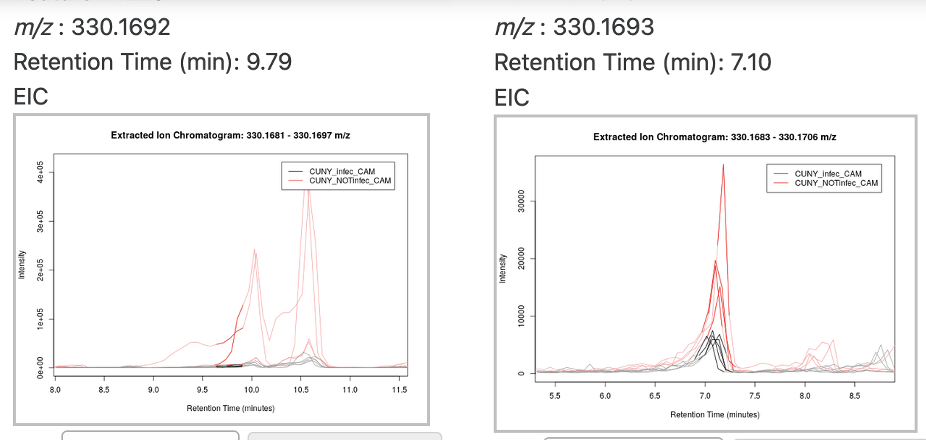


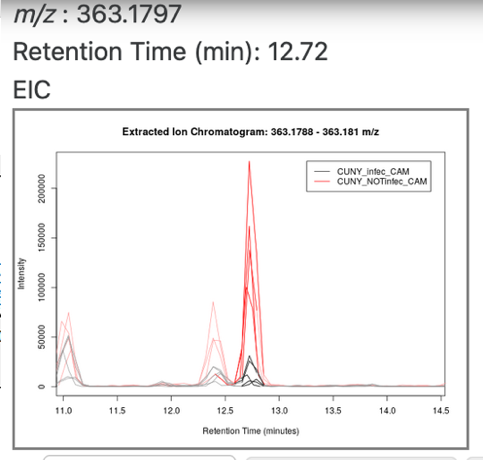

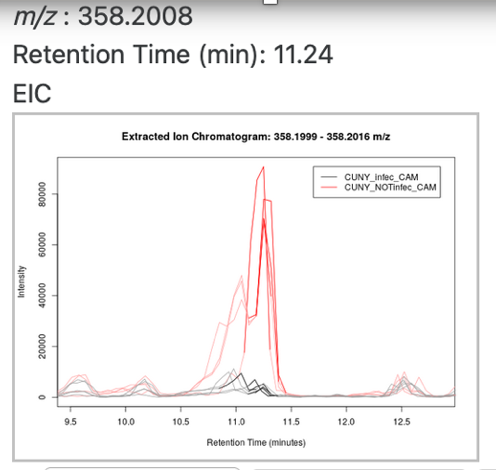


EICs: Downregulated in Non-infected Samples (Red) but upregulated in *Rafflesia*-infected samples (black):


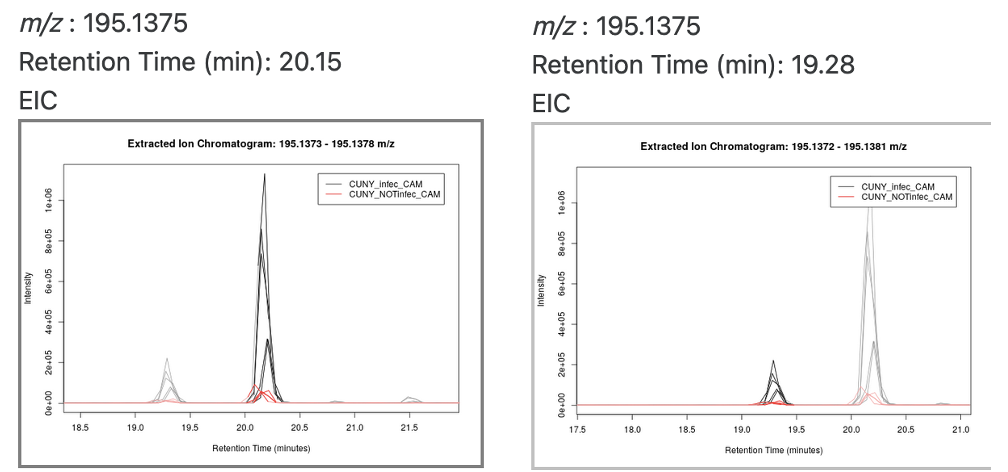

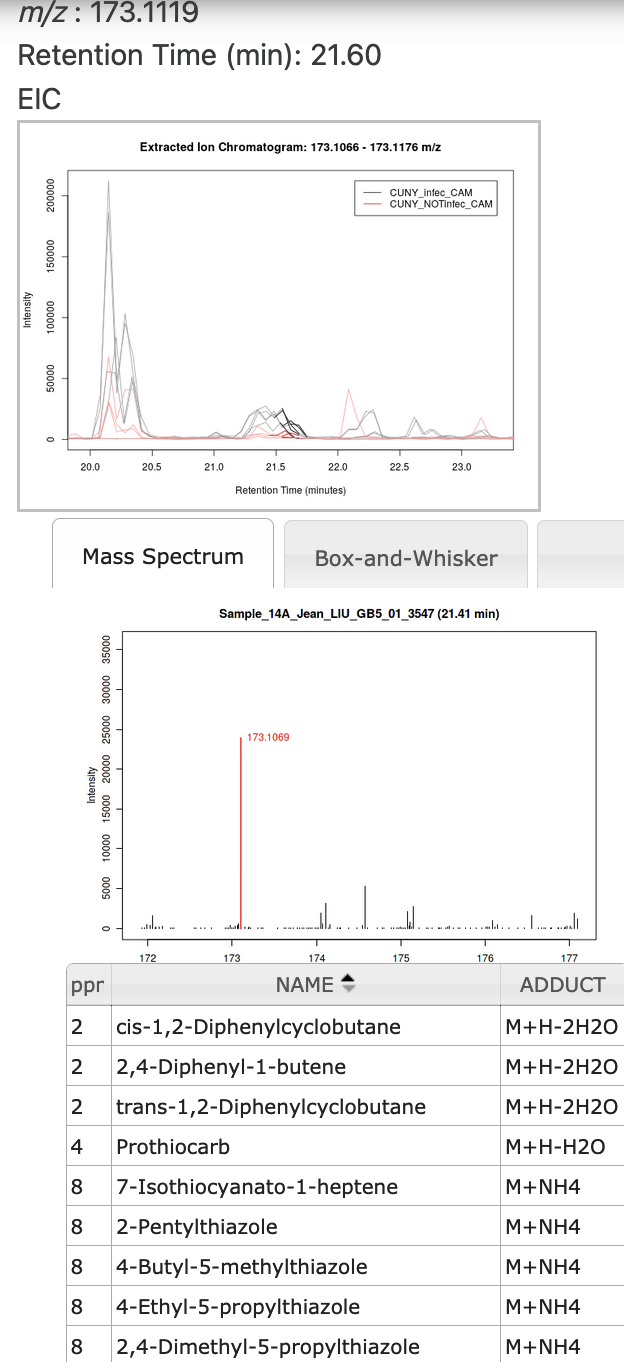


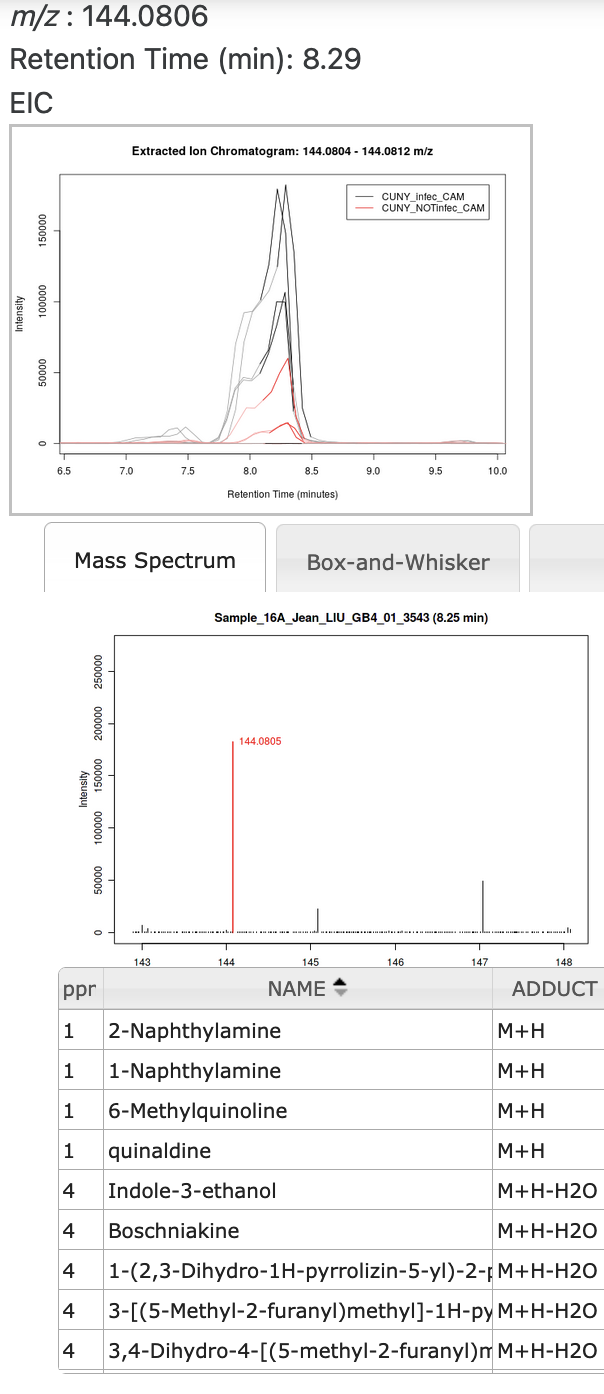


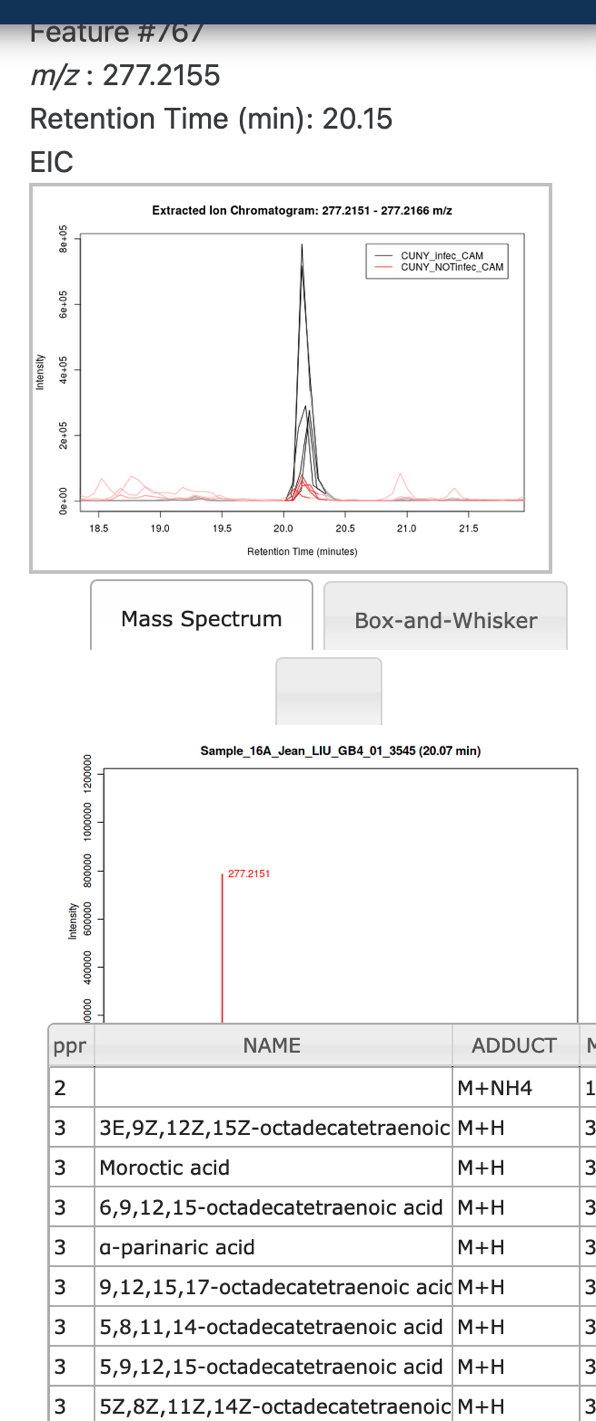


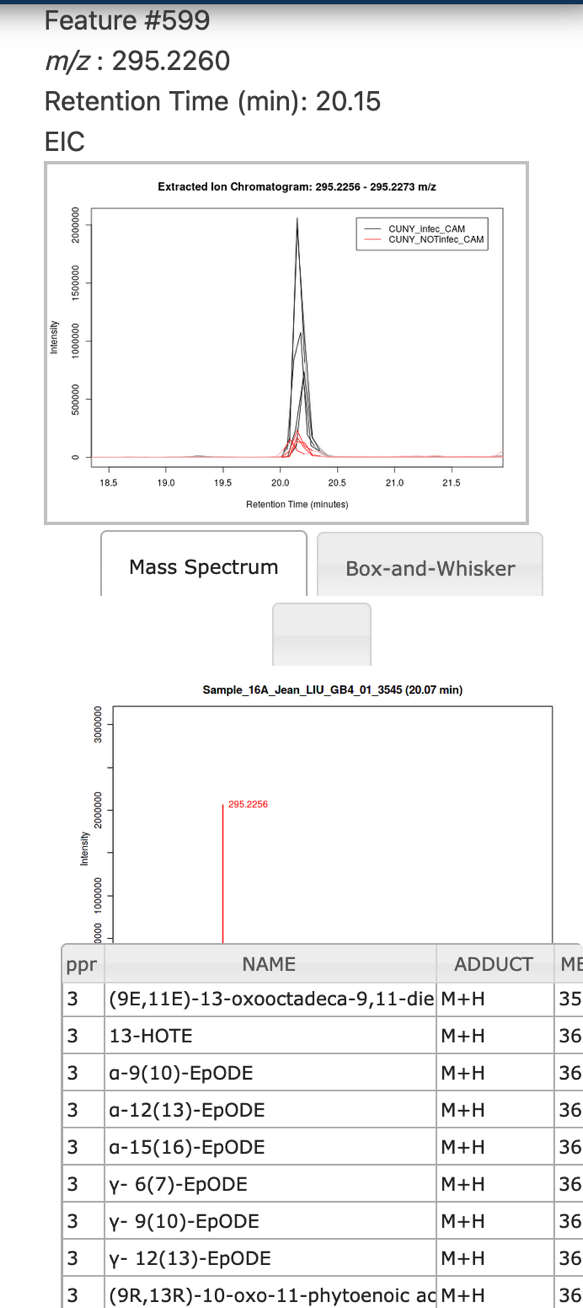

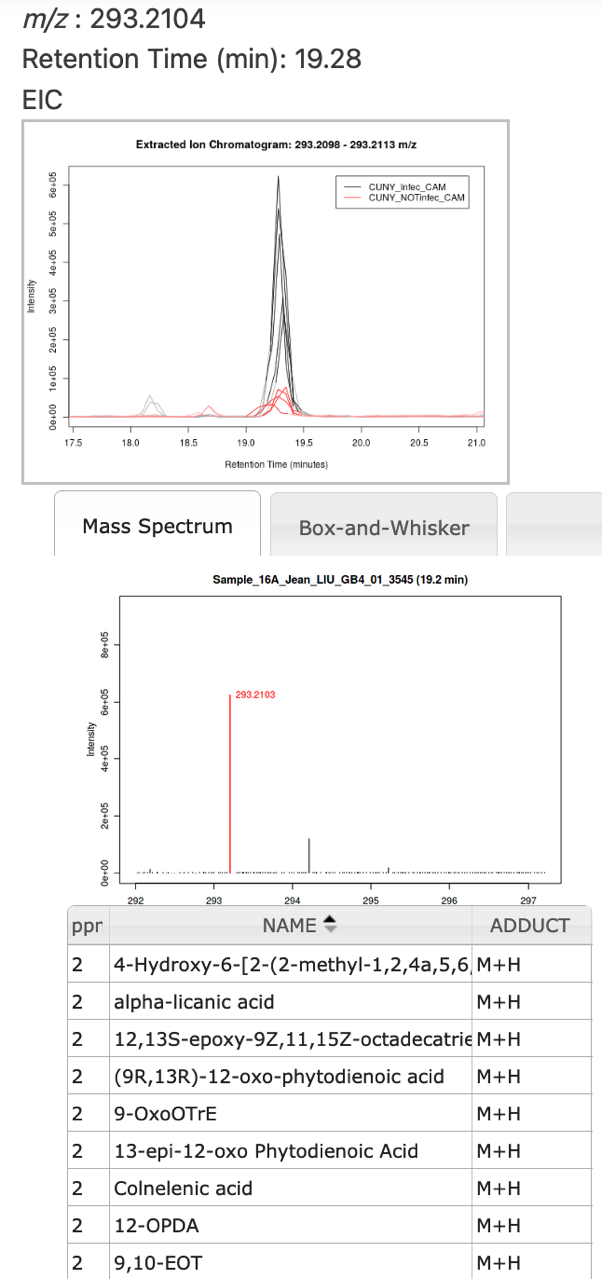


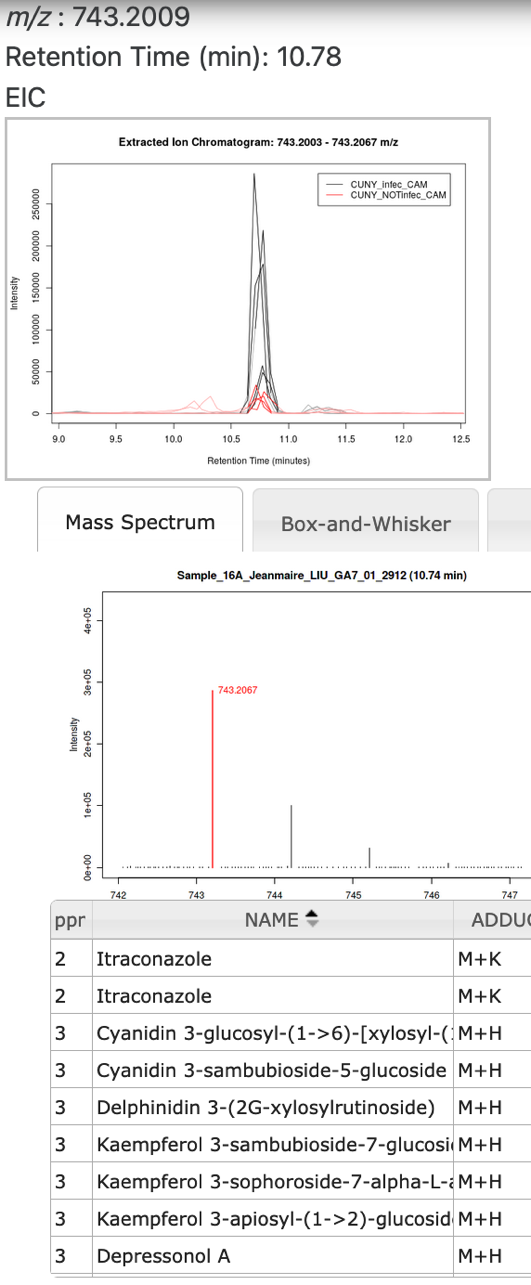

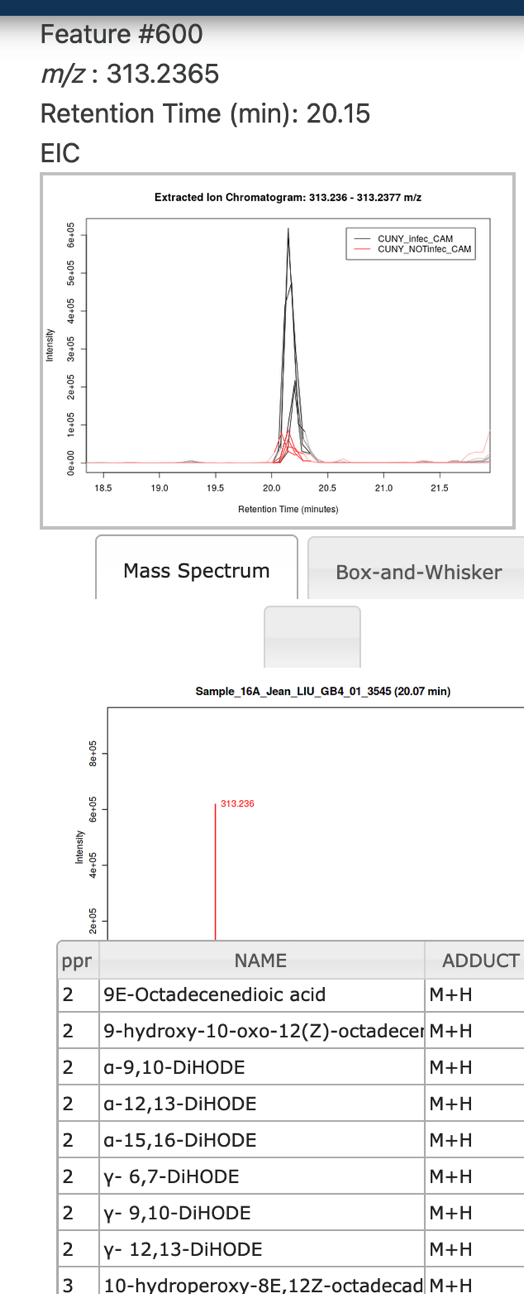

Supplement: Supplementary file 1 — Supplementary file1 (DOCX 2291 kb) [file 425_2021_3787_MOESM1_ESM.docx]
